# Insulin Concentration Modulates Hepatic Lipid Accumulation in Mice in Part via Transcriptional Regulation of Fatty Acid Transport Proteins

**DOI:** 10.1371/journal.pone.0038952

**Published:** 2012-06-20

**Authors:** Samir Softic, Michelle Kirby, Nicholas G. Berger, Noah F. Shroyer, Stephen C. Woods, Rohit Kohli

**Affiliations:** 1 Department of Pediatrics, Division of Gastroenterology, Hepatology, and Nutrition, Cincinnati Children’s Hospital Medical Center, Cincinnati, Ohio, United States of America; 2 Department of Pediatrics, Riley Hospital for Children, Indiana University, Indianapolis, Indiana, United States of America; 3 Division of Developmental Biology, Cincinnati Children’s Hospital Medical Center, Cincinnati, Ohio, United States of America; 4 Metabolic Diseases Institute, Obesity Research Center, Department of Psychiatry and Behavioral Neuroscience, University of Cincinnati College of Medicine, Cincinnati, Ohio, United States of America; The University of Hong Kong, Hong Kong

## Abstract

**Background:**

Fatty liver disease (FLD) is commonly associated with insulin resistance and obesity, but interestingly it is also observed at low insulin states, such as prolonged fasting. Thus, we asked whether insulin is an independent modulator of hepatic lipid accumulation.

**Methods/Principal Findings:**

In mice we induced, hypo- and hyperinsulinemia associated FLD by diet induced obesity and streptozotocin treatment, respectively. The mechanism of free fatty acid induced steatosis was studied in cell culture with mouse liver cells under different insulin concentrations, pharmacological phosphoinositol-3-kinase (PI3K) inhibition and siRNA targeted gene knock-down. We found with *in vivo* and *in vitro* models that lipid storage is increased, as expected, in both hypo- and hyperinsulinemic states, and that it is mediated by signaling through either insulin receptor substrate (IRS) 1 or 2. As previously reported, IRS-1 was up-regulated at high insulin concentrations, while IRS-2 was increased at low levels of insulin concentration. Relative increase in either of these insulin substrates, was associated with an increase in liver-specific fatty acid transport proteins (FATP) 2&5, and increased lipid storage. Furthermore, utilizing pharmacological PI3K inhibition we found that the IRS-PI3K pathway was necessary for lipogenesis, while FATP responses were mediated via IRS signaling. Data from additional siRNA experiments showed that knock-down of IRSs impacted FATP levels.

**Conclusions/Significance:**

States of perturbed insulin signaling (low-insulin or high-insulin) both lead to increased hepatic lipid storage via FATP and IRS signaling. These novel findings offer a common mechanism of FLD pathogenesis in states of both inadequate (prolonged fasting) and ineffective (obesity) insulin signaling.

## Introduction

The incidence of obesity continues to grow, bringing with it an increased prevalence of non-alcoholic fatty liver disease (NAFLD) [1]. While the cause of hepatic steatosis is unknown, obesity-associated hyperinsulinemia is a logical candidate. Nonetheless, the link between insulin and the liver’s handling of lipids is not completely understood and likely is more complex than a simple linear relationship. For instance, NAFLD is also increased in states of low insulin such as poorly controlled type-1 diabetes (T1DM) [2], [3] and prolonged fasting [4], [5].

Insulin receptor substrates (IRS) are well-described intracellular docking molecules that bind to the insulin receptor and initiate the canonical insulin signaling pathway [6]. Findings from knockout mice suggest that most insulin responses associated with nutrient homeostasis are mediated through IRS-1 and/or IRS-2 [7]. IRS-1 is induced in high insulin conditions including the post-prandial state [8] and obesity, whereas IRS-2 is increased in low insulin states such as fasting [9], [10], [11] and caloric restriction [12], [13]. This information, together with the understanding that both high and low insulin states also trigger liver lipid accumulation [2]–[5], begs the question as to whether the relative level of insulin and its concomitant IRS signaling is causally related to the accumulation of lipids in the liver.

To address this question, we focused on fatty acid transport proteins (FATPs) as downstream targets of IRS signaling, since these molecules putatively transport free fatty acids (FFAs) into the cell [14] and are therefore likely intermediaries of hepatic steatosis [15]. Further, FATPs are not exclusively plasma membrane bound and are also found to increase fatty acid transport when present intra-cellularly including on the endoplasmic reticulum and some data suggest that at least in adipocytes FATP1 may translocate to the cell surface from an intracellular perinuclear compartment upon insulin stimulation [16], [17], [18]. Of the various FATPs, FATP-5 is found exclusively in the liver [19], while FATP-2 is found in liver and kidney [20]. Deletion of either FATP-2 [21] or FATP-5 [22], [23], [24] in mice results in decreased hepatic steatosis. In addition, these transporters are up-regulated in the livers of patients with NAFLD [25], [26], [27]. Our overarching hypothesis was that high or low of insulin concentration may trigger the IRS-1 or 2 signaling and consequently activate FATP-2 & 5 mediated fatty acid transport, thus contributing to hepatic lipid accumulation.

## Materials and Methods

### Ethics Statement

All animal studies were approved by the Institutional Animal Care and Use Committee at Cincinnati Children’s Hospital, Cincinnati, Ohio viz. protocol ID number 0D10081.

### Mouse Model of Fatty Liver Disease with Hyperinsulinemia- Type 2 Fatty Liver Disease (T2FLD)

Adult male C57Bl/6 mice (Jackson Laboratory, Bar Harbor, ME) were group-housed 4/cage (22±2°C) on a 12-hour light-dark cycle. Animals were randomized to chow (Teklad-Harlan, Madison, WI), or high-fat high-carbohydrate (HFHC) diet (Surwit diet, 58 kcal % fat; Research Diets, New Brunswick, NJ) and drinking water with high fructose (55% fructose by weight; Acros Organics, Morris Plains, NJ) and sucrose (45% sucrose by weight; Sigma-Aldrich, St. Louis, MO) at a concentration of 42 g/L [28]. Animals were provided ad-lib access to diets for 12 weeks. Body weights and food intake were recorded.

### Mouse Model of Fatty Liver Disease with Hypoinsulinemia- Type 1 Fatty Liver Disease (T1FLD)

The same strain, age, and gender of mice were utilized under identical conditions as mice in T2FLD model. Mice were kept on chow or high-fat diet (HFD; 60% kcal predominantly palmitic, stearic and oleic fatty acids -Research Diets, New Brunswick, NJ) for 2 weeks. After two weeks a group of chow and HFD mice were injected ip with 200 mg/kg Streptozotocin (Sigma-Aldrich, St. Louis, MO), dissolved in Citric acid buffer, pH = 4.2. Mice were sacrificed three days after STZ injection.

### T1FLD (Mouse Model of Fatty Liver with Hypoinsulinemia) Mice Treated with Exogenous Insulin Replacement

Mice were treated as in T1FLD model with following exceptions. Three days after STZ injection blood glucose was measured after 4 hour fast (Roche Diagnostics, Indianapolis, IN). Animals in HFD, STZ treated group with glucose values >250 mg/dl were assigned into three groups. Group 1 was i.p. injected with normal saline (NS) 200 µl/mouse, group 2 received 20, while group 3 received 200 mU/mouse of insulin (Lantus™, Sanofi-Aventis, Bridgewater, NJ), dissolved in 200 µl of NS. After insulin injection mice were sacrificed after a 12 fast.

### Plasma Free Fatty Acids

12 hour fasting plasma samples from T1FLD mice treated with exogenous insulin replacement were analyzed for total free fatty acid content using a weighed sample of 100 µL of plasma and 10 µg of heptadecanoic acid in hexane solution. Samples were saponified with methanolic NaOH and methylated with BF_3_ in methanol according to the method of Metcalfe et al. [29]. Samples were extracted from the aqueous phase and then analyzed by gas chromatography as described earlier. [30].Fatty acids were identified and total mass quantified based on the mass of the added heptadecanoic acid standard.

### Histology

Harvested livers were submitted to the Department of Pathology at Cincinnati Children’s Hospital Medical Center for hematoxylin & eosin (H&E) staining and preparation of frozen sections.

### Cell Culture Model of Steatosis

We used a previously established cell culture model of hepatic steatosis induced by feeding 1 mM (2∶1) oleate (Sigma-Aldrich, St. Louis, MO)/palmitate (Sigma-Aldrich, St. Louis, MO) mixture to TGF-α immortalized mouse hepatocytes (AML12 cells, ATCC, Manassas, VA) [31]. In brief, cells were grown for 48 hours, made quiescent by growing them in cell-culture media without fetal bovine serum for 24 hours and subsequently treated with different concentrations of insulin (0, 2.5, 10, 20, 50 and 100 mU/ml {100 mU/ml being equivalent to 0.69 mM or 4.0 µg/ml}) for 24 hours, then harvested after FFA (1∶100 dilution) exposure for 12 hours. In some experiments, 50 uM LY294002 (PI3K inhibitor) was added to AML12 cells 12 hours prior to addition of FFA enriched medium.

### Oil Red-O Staining

Cultured hepatocytes grown in 12 well plates on glass cover slips were stained using oil red-O kit (American MasterTech, Lodi, CA). Similar staining was performed on T2FLD and T1FLD liver frozen sections.

### Triglyceride (TG) and Alanine Amino Transferase (ALT) Quantification

Intracellular TG content was determined from harvested cells and/or mouse liver homogenates as previously described [32]. Briefly, 100 milligrams of wet liver tissue or a monolayer of plated cells were homogenized in a 20 mM Tris buffer. Triglyceride reagent set (Pointe Scientific, Canton, MI) was used for the assay per manufacturer’s instructions. Photometric absorbance was read at 500 nm using Synergy 2 microplate reader (BioTek, Winooski, VT). ALT was measured from cell supernatant or mouse serum utilizing DiscretPak™ ALT Reagent Kit (Catachem, Bridgeport, CT). ALT enzyme kinetics was measured over a five minute interval by measuring change in photometric absorbance at 340 nm.

### qPCR Gene Expression

RNA was isolated from cultured cells and/or frozen liver tissue. Initially, the samples were homogenized in a 20 mM Tris buffer. Total RNA was isolated using TRIzol® reagent protocol (Molecular Research Center, Cincinnati, OH). cDNA was made using TaqMan™ Reverse Transcription protocol (Applied Biosystems, Carlsbad, CA) on an Eppendorf Mastercycler PCR machine (Eppendorf North America, Westbury, NY). Gene specific primer sequence 5′ to 3′ ends is as follows: GAPDH forward TGGTTTGACAATGAATACGGCTAC reverse GGTGGGTGGTCCAAGGTTTC, FATP-2 forward ACTGCTCAAACTGGGCTGTC reverse TGGAAGAACCTCCTCCACAG, FATP-5 forward ACTCTGTACGGAGCTCTGGG reverse GAGCTGTGGCCAAGGTAGAA, IRS-1 forward GCCAGAGGATCGTCAATAGC reverse AGGTCCTGGTTGTGAATTGTG, IRS-2 forward TCCGCGGCTGGAGTACTACGAG reverse ACAGCAGTCGAGCGCGATCAC.

Messenger RNA expression was measured using Stratagene SYBR® green real-time kinetic PCR on a Stratagene Mx-3005 Multiplex Quantitative PCR machine (Stratagene, Agilent Technologies, La Jolla, CA). The standard curve method was utilized to calculate relative gene expression.

### RNAi Cell Culture Experiments

AML-12 cells were grown to 80% confluence as above, then transfected according to manufacturer’s protocol. In brief, Opti-MEM® (Invitrogen, Carlsbad, CA) at a final volume of 250 µL each was used to prepare Lipofectamine™ 2000 (Invitrogen, Carlsbad, CA) and IRS1 & 2, FATP 2 & 5, small interference (si) RNA in 6 well plates. The complex was added to the well with 1.5 mL of medium without antibiotics and incubated at 37°C for 12 hours. siRNA oligomers (Invitrogen, Carlsbad, CA) used were: IRS1, sense sequence GGUCAGACAAAGAACCUGAtt and antisense sequence UCAGGUUCUUUGUCUGACCca; IRS2, sense sequence UCAUGUCCCUUGACGAGUAtt and antisense sequence UACUCGUCAAGGGACAUGAaa; FATP2 (Slc27a), sense sequence CAUCAAUCAUCAUCGCCUAtt and antisense sequence UAGGCGAUGAUGAUUGAUGgt; FATP5 (Slc27a5), sense sequence GCUACGAUUAAGUGGAAAUtt and antisense sequence AUUUCCACUUAAUCGUAGCtc. Following transfection, the media was changed and the cells were made quiescent for 24 hours and subsequently fed FFA for 12 hours. Following purification of total RNA and synthesis of cDNA, real-time PCR analysis was performed with pre-designed, validated gene-specific TaqMan® probes (Applied Biotechnology, Carlbad, CA). Relative expression was determined by normalizing to GAPDH expression. Primer probe sets used to evaluate gene knockdown on gels were: Mm01278327_m1(IRS1); Mm03038438_m1(IRS2); Mm00449517_m1(FATP2); Mm00447768_m1(FATP5); Mm99999915_g1(GAPDH).

### FATP-2 Immunofluorescence

Cells were grown on cover-slips and treated as outlined above in the *in vitro* cell culture steatosis model. Thereafter they were fixed in ice cold acetone, blocked in 5% BSA solution and stained with primary antibody against FATP-2 (Abcam, Cambridge, MA) and secondary anti-mouse FITC antibody (Abcam, Cambridge, MA). Images were taken on a Zeiss epifluorescence microscope (Zeiss, Pleasanton, CA).

### Statistical Analysis

Results are expressed as mean ± SEM. Where indicated, the statistical significance between two groups was estimated by Student’s t test or among three or more groups using ANOVA. *, **, *** and indicate statistical significance with p<0.05, p<0.01 and, p<0.001 respectively. Statistically non-different results were labeled ns where appropriate.

## Results

### *In vivo* Models of Streptozotocin Induced Hypoinsulinemia (T1FLD) and Diet Induced Hyperinsulinemia (T2FLD) both Result in Hepatic Steatosis

To test the effect of hypoinsulinemia on liver TG accumulation we injected mice, that had been fed a HFD for 2 weeks, with streptozotocin to ablate insulin producing pancreatic β-cells i.p. as described before [33] (T1FLD). To address the effect of hyperinsulinemia on liver TG accumulation, we placed mice on a HFD for 12 weeks as previously described [28] (T2FLD).

**Figure 1:**
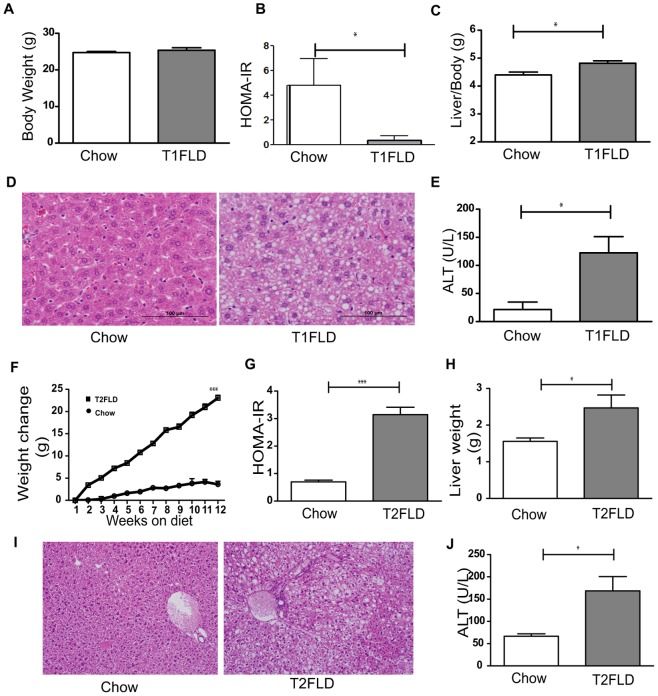
*In vivo* model of hypoinsulinemia on high-fat diet leads to fatty liver disease. (**A**) Weight in grams after two weeks of chow (Chow) or high-fat diet, followed by streptozotocin injection (T1FLD). (**B**) HOMA-IR at sacrifice (**C**) Liver to body weight ratio. (**D**) H&E stain of representative liver sections and (**E**) serum ALT levels. *In vivo* model of hyperinsulinemia on high-fat diet leads to fatty liver disease. (**F**) Weight change of chow (Chow) and high fat diet fed mice (T2FLD) after 12 weeks on respective diets. (**G**) HOMA-IR, a measure of insulin resistance and (**H**) liver weight from the same mice. (**I**) H&E stain of representative liver sections and (**J**) serum ALT levels. [*P<0.05, **P<0.01 & ***P<0.001, n = 4–6 for chow, 5 for T1FLD and 6 for T2FLD. Representative of two-three replicate experiments.]

**Figure 2:**
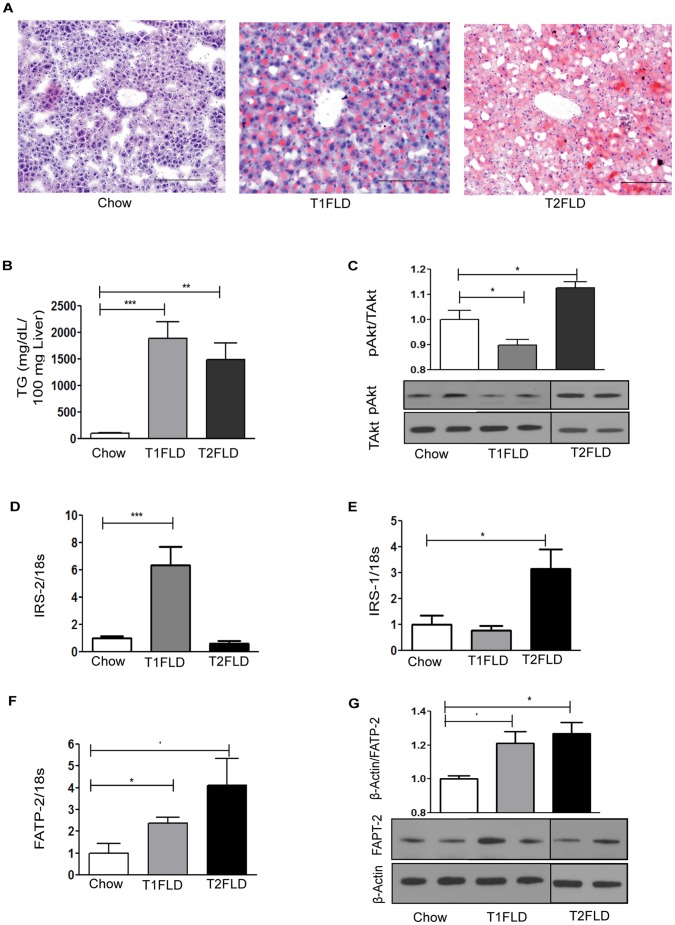
*In vivo* models of hypoinsulinemia on HFD (T1FLD) and hyperinsulinemia on HFD (T2FLD) lead to fatty liver disease. (**A**) Oil red-O staining (red) of representative liver sections scale bar = 100 µm of chow fed(Chow), mice fed HFD for 2 weeks and then streptozotocin injected for three days (T1FLD) and HFD fed mice for 12 weeks (T2FLD). (**B**) TG content per 100 mg of liver tissue in Chow, T1FLD, and T2FLD mice (**C**) Liver pAkt of Chow, T1FLD, and T2FLD mice (**D**) Liver IRS2 mRNA expression normalized to 18s ribosomal protein in Chow, T1FLD, and T2FLD, (**E**) Liver IRS1 mRNA expression normalized to 18s ribosomal protein in Chow, T1FLD, and T2FLD (**F**) Liver FATP2 mRNA expression normalized to 18s ribosomal protein in Chow, T1FLD, and T2FLD, (**G**) Liver FATP2 protein on Western Blot normalized to β-actin in Chow, T1FLD, and T2FLD, [One way ANOVA with Tukey’s post-hoc test *P<0.05, **P<0.01 & ***P<0.001; n = 5–8 per group, representative of three replicate experiments.]

At sacrifice, T1FLD mice weighed the same as controls (Figure 1A), but had developed fasting hyperglycemia and hypoinsulinemia resulting in a significant reduction in HOMA-IR (Figure 1B). Despite their unchanged body weight these hypoinsulinemic mice had increased liver to body weight ratio (Figure 1C), H&E staining indicative of steatosis (Figure 1D) and increased serum ALT (Figure 1E) relative to chow fed-saline injected controls. This was further matched by increased oil red-O staining (Figure 2A) and liver TG accumulation (Figure 2B). Interestingly, the STZ injected T1FLD mice had hepatic steatosis but with low circulating plasma insulin levels they had a reduced liver pAkt to total Akt ratio (Figure 2C).

In comparison when T2FLD were sacrificed they had gained more weight than chow-fed control mice (Figure 1F), and had developed fasting hyperglycemia, and hyperinsulinemia, resulting in a significant increase in HOMA-IR (Figure 1G).These mice also had increased liver weight (Figure 1H), H&E staining indicative of steatosis (Figure 1I), and increased serum ALT (Figure 1J). Furthermore, these mice had increased steatosis on Oil Red-O (Figure 2A) and increased intracellular TGs (Figure 2B). This high-fat diet fed murine model which induced hepatic steatosis with hyperinsulinemia, was also associated with increased liver pAkt to total Akt ratio by western blotting (Figure 2C).

### Increased IRS-2 in T1FLD and Increased IRS-1 in T2FLD are Associated with Augmented FATP Expression and Protein Levels

Hypoinsulinemic T1FLD mice had increased levels of IRS-2 hepatic gene expression, which was absent in T2FLD mice (Figure 2D). Conversely, liver IRS-1 mRNA expression levels were increased in T2FLD mice, without significant change in T1FLD mice (Figure 2E).

Both T1FLD and T2FLD mice livers had an increase in FATP-2 mRNA (Figure 2F) and protein levels (Figure 2G), as well as FATP-5 mRNA expression (Data not shown). Thus, *in vivo* hypoinsulinemic T1FLD mice developed hepatic steatosis, which was associated with an up-regulation of IRS-2 relative to IRS-1, and an increase in FATP-2 and FATP-5 mRNA expression, as well as greater FATP-2 protein levels.

Conversely, *in-vivo* HFD induced hyperinsulinemic T2FLD mice were associated with an up-regulation of IRS-1 relative to IRS-2 (Figure 2 D and E), and an increase in FATP-2 (Figure 2 F) and FATP-2 protein (Figure 2 G), as well as greater FATP-5 mRNA expression (Data not shown).

### Insulin Replacement to Hypoinsulinemic Mice on HFD (T1FLD) Reduces Fatty Liver

**Figure 3:**
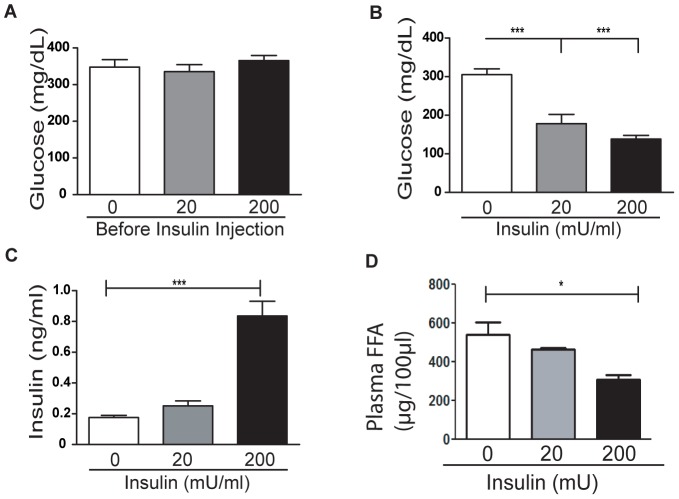
Insulin replacement to hypoinsulinemic mice (T1FLD) reduces plasma glucose and free fatty acids (FFA). (**A**) Fasting blood glucose of T1FLD mice generated as before (Figure 1 and 2) (**B**) and 12 hours after incremental Lantus replecement (0, 20 & 200 mU/mouse). (**C**) Plasma insulin and (D) FFA levels following Lantus treatment were reduced. [***P<0.001, n = 7 per group, representative of one experiment.]

When considered together, the data from our two *in vivo* mouse models indicate that either high or low insulin levels lead to steatosis, while normoinsulinemic mice do not develop excessive liver lipid accumulation. To determine whether insulin levels *in vivo* can modulate liver lipid accumulation, we treated a group of hypoinsulinemic T1FLD mice with increasing doses of insulin (Lantus ™) for 12 hours. As before, prior to insulin replacement the mice were hyperglycemic (Figure 3A). This hyperglycemia was reversed by insulin replacement in a dose-dependent manner (Figure 3B) resulting in progressively elevated insulin levels (Figure 3C). Plasma free fatty acid levels were also decreased in a dose dependent manner with increasing insulin administration (Figure 3D).

**Figure 4:**
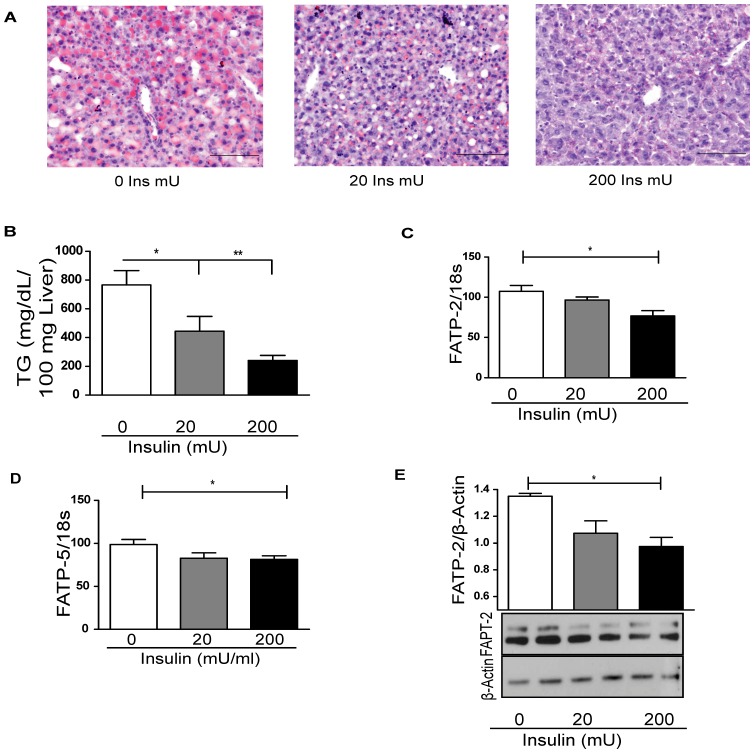
Insulin replacement to hypoinsulinemic mice (T1FLD) reduces fatty liver. (**A**) Oil Red-O staining (red) and hematoxylin (blue) of representative liver sections, scale bar = 100 µm for T1FLD provided with Lantus™ replacement (0, 20 & 200 mU/mouse) for 12 hours and (**B**) TG content per 100 mg of liver tissue in T1FLD provided with Lantus™ replacement (0, 20 & 200 mU/mouse) for 12 hours. (**C**) Liver FATP-2 mRNA expression in T1FLD mice with Lantus™ replacement (0, 20 & 200 mU/mouse) for 12 hours. (**D**) Liver FATP-5 mRNA expression in T1FLD mice with Lantus™ replacement (0, 20 & 200 mU/mouse) for 12 hours. (**E**) Liver FATP-2 western blot normalized to β-actin in T1FLD mice with Lantus™ replacement (0, 20 & 200 mU/mouse) for 12 hours. [One way ANOVA with Tukey’s post-hoc test *P<0.05, **P<0.01, ***P<0.001, n = 5–6 per group.]

The livers of insulin-treated mice had a dose-dependent decrease in steatosis by oil red-O (Figure 4A). This was also reflected in liver TG quantification (Figure 4B). FATP-2 (Figure 4C) and FATP-5 (Figure 4D) mRNA expression levels were decreased after insulin treatment, as was FATP-2 liver protein (Figure 4E). Thus exogenous insulin decreased both circulating FFA and liver fatty acid transport related proteins. This experiment confirmed that liver TG accumulation in T1FLD mice can be reversed by exogenous insulin and it is associated with the down regulation of FATP-2 and FATP-5 i.e., reversal of the insulin deficiency resulted in reversal of liver TG accumulation.

### Insulin Modulates Triglyceride Accumulation and FATP Expression *in vitro*

Hepatocytes were cultured in the presence of basal levels of insulin and fed FFA. Relative to control cells, FFA-enriched cells had increased oil red-O staining (data not shown), as well as increased intracellular TG content (data not shown) and supernatant ALT (data not shown). These findings were consistent with our and other previous reports [31], [34].

**Figure 5:**
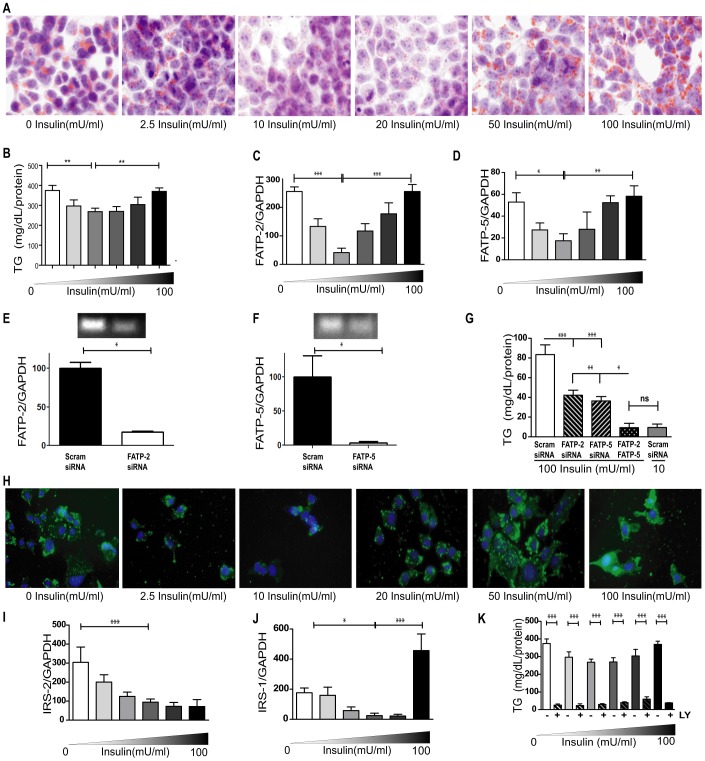
Insulin mediates triglyceride accumulation and FATP expression *in vitro via* IRS-1 and IRS-2 expression. (**A**) Oil Red-O stain (red) for lipids and hematoxylin nuclear stain (blue) of AML-12 hepatocytes fed FFA for 12 hours in varying insulin concentrations (0, 2.5, 10, 20, 50 and 100 mU/ml). Original magnification × 400. (**B**) Intracellular TG quantification as well as (**C**) FATP-2 & (**D**) FATP-5 mRNA expression of hepatocytes. (**E**) qPCR (bar graph) results of targeted gene knockdown using scrambled and FATP2 siRNA at 50 pmol concentration; efficient knockdown was confirmed by running PCR reactions on an agarose gel. (**F**) qPCR (bar graph) results of targeted gene knockdown using scrambled and FATP5 siRNA at 50 pmol concentration; efficient knockdown was confirmed by running PCR reactions on an agarose gel. (**G**) TG quantification following targeted gene knock-down against Scrambled, FATP-2, FATP-5, or both FATP-2 & FATP-5. (**H**) Immunofluorescently labeled FATP-2 protein (green) counter stained with DAPI (blue) nuclear stain treated as in panel A. (**I**) IRS-2 & (**J**) IRS-1 mRNA expression of AML-12 hepatocytes fed FFA for 12 hours in varying insulin concentrations (0, 2.5, 10, 20, 50 and 100 mU/ml). (**K**) TG accumulation of FFA fed cells with (+) or without (−) 50 uM LY294002 (LY). Data for without LY also shown in Fig 4B. [ANOVA *P<0.05, **P<0.01 & ***P<0.001; n = 6 per group, representative of three replicate experiments.]

Variation of insulin concentration in the above setting resulted in a U-shaped bimodal function of positive oil red-O staining (Figure 5A), that paralleled hepatic TG accumulation (Figure 5B). The most intense staining and peak hepatocyte TG content occurred at the lowest and highest concentrations of added insulin (0 and 100 mU/ml, respectively), while the nadir was observed at 10 mU/ml. For cells incubated in the absence of added FFA, there was no dose-effect TG accumulation in response to added insulin alone (data not shown). Further, supernatant glucose levels were not changed for insulin-treated cells (data not shown), in agreement with previous reports indicating that insulin does not stimulate glucose uptake in the liver [35]. These data suggest that while insulin elicits a dose-dependent and FFA-dependent TG accumulation in the liver, glucose utilization is unlikely to mediate its U-shaped effect.

To assess whether insulin-mediated FFA transport may be a factor, we measured the expression of FATPs *in vitro*. A comparable bimodal insulin-response curve occurred for FATP-2 (Figure 5C) and FATP-5 (Figure 5D) mRNA, as well as for FATP-2 protein immunofluorescence (Figure 5F). We used siRNA against FATP-2 & 5 and observed an almost 50% reduction in TG accumulation with either FATP-2 or 5 knock-down (Figure 5E). Simultaneous knock-down of both FATP-2 & 5, resulted in an almost complete normalization of hepatocyte TG levels. These experiments further confirmed that FATP expression is sensitive to insulin, and that the consequent U-shaped function of TG accumulation mimics a change in FATP-2 & 5 mRNA levels.

### *In vitro* IRS-1 and IRS-2 Expression is Dependent on Insulin Concentration

Our *in vivo* studies suggest that IRS substrates may be differentially expressed at extremes of insulin concentrations. Thus, IRS expression was again assessed in cell culture model of steatosis and increased IRS-2 (Figure 5G) mRNA levels were found at low insulin concentrations, while our highest insulin condition resulted in increased IRS-1 (Figure 5H) mRNA. In addition, a small but significant rise in IRS-1 was also observed at low insulin conditions however this *in vitro* increase in IRS-1 at low insulin levels was not observed in our *in vivo* studies. To further test whether changes in IRS expression are implicated in lipogenesis, we inhibited its downstream target PI3K with LY294002 (LY). A marked reduction in TGs was seen with LY, regardless of insulin concentration (Figure 5I). This suggested that TG accumulation in both hypo and hyperinsulinemia converges onto a common PI3K dependent pathway.

### *In vitro* Inhibition of IRS Mediated FATP Expression Leads to Decreased TG Accumulation

**Figure 6:**
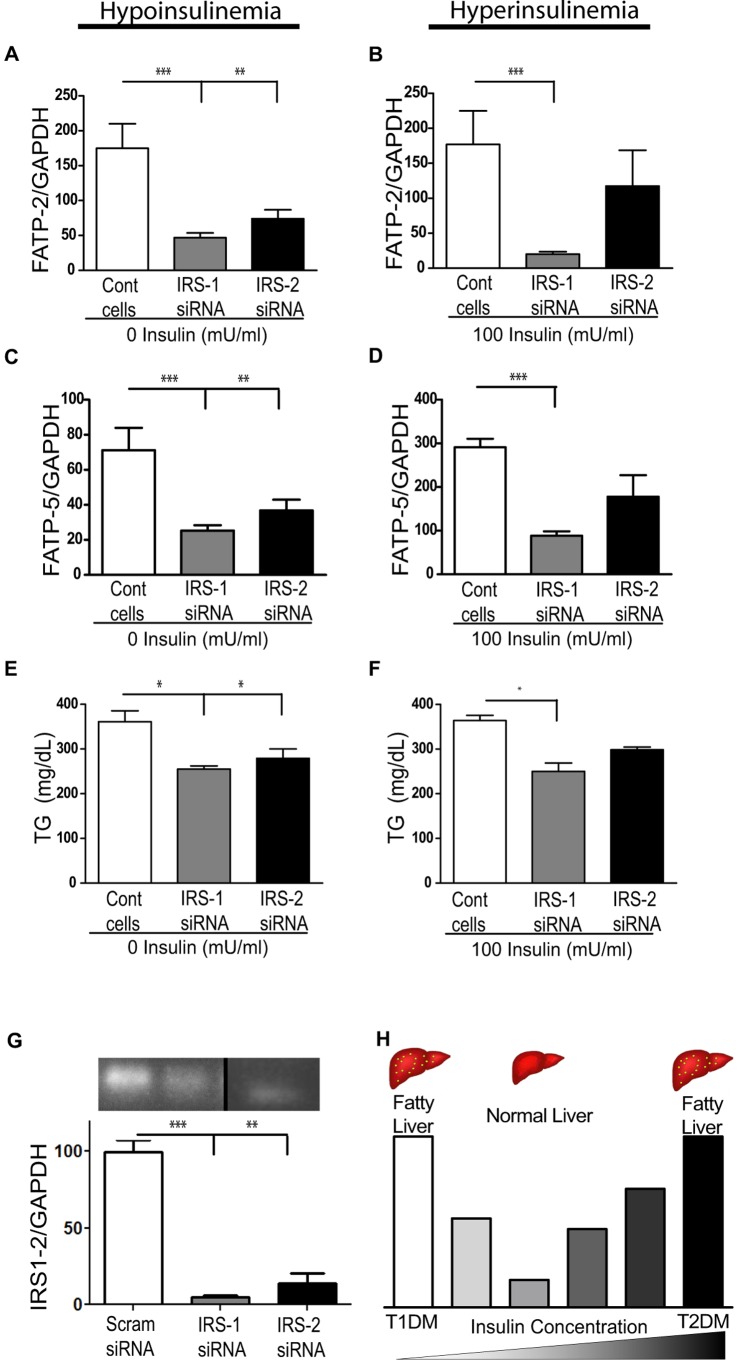
*In vitro* inhibition of IRS mediated FATP expression leads to decreased TG accumulation. FATP-2 mRNA after IRS-1 or IRS-2 siRNA knock-down at 0 (**A**) and (**B**)100 mU/ml of insulin. (**C&D**) FATP-5 mRNA expression under identical conditions. TG accumulation after IRS-1 or 2 siRNA knock-down at 0 (**E**) and (**F**) 100 mU/ml of insulin. (**G**) qPCR (bar graph) results of targeted gene knockdown using scrambled and IRS1 and IRS2 siRNA at 15 and 60 pmol concentration, respectively; efficient knockdown was confirmed by running PCR reactions on an agarose gel. (**H**) Illustration of insulin mediated bimodal response leading to steatosis. [ANOVA *P<0.05, **P<0.01 & ***P<0.001; not significant (ns), n = 3 per group, representative of three replicate experiments.]

A stronger link between IRSs and FATPs was further established in experiments using siRNA inhibition of IRS-1 & 2. FATP-2 mRNA expression at 0 mU/ml insulin decreased after both IRS-1&2 knock-down, while at 100 mU/ml insulin only IRS-1 knock-down resulted in a significant decrease in FATP-2 mRNA (Figure 6A & B). Similarly, FATP-5 mRNA at 0 mU/ml insulin decreased after both IRS-1 & 2 knock-down, while at 100 mU/ml insulin only IRS-1 knock-down resulted in a significant decrease in FATP-5 mRNA (Figure 6C & D). Analogous to this decrease in FATPs, IRS-1 & 2 knock-down at 0 mU/ml insulin resulted in a decrease in hepatocyte TG content, while only IRS-1 knock-down at 100 mU/ml insulin resulted in a decrease in hepatocyte TG accumulation (Figure 6E & F). Thus, inhibition of IRS-1 or IRS-2 at states of their respectively augmented expressions leads to decreased FATP expression and subsequent reduction in hepatic TG accumulation.

## Discussion

Our *in vivo* models of hepatic steatosis with perturbed insulin concentrations had relatively increased IRS-1 expression at high insulin concentrations (T2FLD), and relatively increased IRS-2 at low insulin concentrations (T1FLD) (Figure 6G). This relative ‘imbalance’ between the two IRS molecules was associated with an up-regulation of FATP-2 & 5 in both states. Holding other parameters constant *in vitro,* we replicated this bimodal TG accumulation function simply by varying insulin levels. We thus describe a novel insulin driven bimodal FATP-lipid accumulation response.

FATP (later named FATP1) was first identified in 1994 by Schaffer et al and shown to increase the uptake of long chain fatty acids across the plasma membrane [36]. The murine *Fatp1* gene was found to span approximately 16 kilobases and contain 13 exons, of which exon 2 was shown to be alternatively spliced. Since then multiple groups have worked on various isoforms of FATP in different tissues of interest. Further, human relevance has been described and researched in the setting of X-linked adrenoleukodystrophy, a genetic neurodegenerative disorder wherein increased levels of saturated very long-chain fatty acids are found in tissues and plasma. Herein FATP2 has independently been identified as a hepatic peroxisomal very long-chain acyl-CoA synthetase [21], [37]. Further, in adipocytes *FATP1* has been shown to be transcriptionaly regulated by insulin [38]. Thus, given the putative role of FATPs in hepatic lipid transport and their potential to be regulated by insulin in other tissues we set about looking into the link between insulin and lipid transport and its relation to FATP 2 and 5 in the liver.

Investigating the hyperinsulinemic state Kerouz *et al*. have reported that obese ob/ob mice have significantly higher IRS-1 than IRS-2 liver protein [39]. Furthermore, Guo *et al*. reported that inactivation of IRS-1 leads to improvement in murine hepatic steatosis [40]. Further, Taniguchi *et al*. found that short term adenovirus mediated inactivation of IRS-2 increased hepatic steatosis [41]. Thus, we conclude from the literature our data that an imbalance of IRS signaling favoring relatively more IRS-1 than IRS-2 occurs in the hyperinsulinemic state.

Investigating the hypoinsulinemic state Rojas *et al*. observed that liver IRS-2 protein increased relative to IRS-1 after a 72-hour fast or with STZ-induced T1DM [42]. Contrary to these findings, Simmgen *et al*. reported that IRS-2 signaling is not required for hepatic lipid metabolism [43]. However, the authors of these studies did not assess this in either fasting or STZ-induced conditions, where IRS-2 is shown to be increased. We observed in our insulin replacement experiment a dose dependent reduction in liver TG content in STZ treated T1FLD mice receiving insulin. Interestingly we also observed a significant decrease in fasting FFA plasma levels associated with increasing exogenous insulin doses. Though we did not measure fatty acid transport directly in our current experiments this is clearly an important path of future research. This decrease in FFA maybe a manifestation of direct insulin mediated reduction in peripheral lipolysis and therefore contribute to the reduction in TG accumulation independent of FATP regulation.

We acknowledge that these two *in vivo* models may not be completely translatable to human conditions such as type 2 and type 1 diabetes, however, further evidence that insulin impacts the FATP levels directly comes from our *in vitro* studies where targeted gene disruption of IRS-1&2, led to decreased FATP-2&5 expressions. Though all *in vitro* experiments were not performed under identical conditions (siRNA knockdown required an additional 12 hours of incubation, resulting in a different magnitude of TG accumulation) unlike *in vivo*, there is no peripheral lipolysis *in vitro,* thus we concluded that increased FATP expression at extremes of insulin concentrations is likely mediated via imbalanced IRS signaling. We acknowledge that some of the concentrations of insulin used (e.g. 100 mU/ml) maybe be supraphysiological but they do provide for proof of concept.

Indeed, others have also found that IRS-1 mRNA and protein levels are increased with insulin treatment [8], and that IRS-2 mRNA is down regulated by insulin [44]. Thus, if a relative increase of IRS-1 signaling is paramount in the pathogenesis of obesity comorbidities, then a pharmacologic means of restoring IRS-2 signaling might prove to be a viable therapeutic option. White *et al* have reported that finding drugs which stimulate IRS-2 synthesis or promote its signaling might be a useful treatment option for obesity-associated T2DM [45]. Similarly, Gupta *et al.* have reported that the long-acting glucagon-like peptide 1 (GLP-1) agonist, Exendin-4, decreases hepatic steatosis and activates the same pathway as IRS-2 [46]. Also of note, insulin has been shown in other tissues, such as cardiac myocytes and adipocytes, to increase fatty acid uptake [18], [47]. Furthermore, drugs used to treat T2DM, which also improve hepatic steatosis, such as rosiglitazone [48], [49] and metformin [50], are found to preferentially increase and restore IRS-2 expression.

The main finding of this report is that the amount of circulating insulin is a major modulator of hepatic steatosis via regulation of liver fatty acid transport proteins. In both *in vitro* and *in vivo* experiments, insulin-mediated TG accumulation in the liver exhibited a bimodal function, where both hypo and hyperinsulinemia led to augmented liver fat storage. While we observed reduced FATP 2 and 5 in these experiments, the reduction in TG levels could also be attributable to enhanced fatty acid oxidation, decreased de novo lipogenesis or altered lipoprotein export. These pathways definitely need to be evaluated in future experiments in order to understand the contribution of altered FFA uptake. Ultimately, the sums of all of these processes appear to be mediated via an imbalance of insulin substrates, where hypoinsulinemia is characterized by excessive IRS-2 signaling, and hyperinsulinemia with predominant IRS-1 signaling. Regardless as to which side the equilibrium is shifted; imbalanced insulin signaling points to a common potential FATP response. The identification and verification of this novel link in follow up experiments may provide future therapeutic targets for the treatment of obesity associated fatty liver disease.

## References

[1] Angulo P (2002). Nonalcoholic fatty liver disease.. N Engl J Med.

[2] Leeds JS, Forman EM, Morley S, Scott AR, Tesfaye S (2009). Abnormal liver function tests in patients with Type 1 diabetes mellitus: prevalence, clinical correlations and underlying pathologies.. Diabet Med.

[3] Targher G, Bertolini L, Padovani R, Rodella S, Zoppini G (2010). Prevalence of non-alcoholic fatty liver disease and its association with cardiovascular disease in patients with type 1 diabetes.. J Hepatol.

[4] Kersten S, Seydoux J, Peters JM, Gonzalez FJ, Desvergne B (1999). Peroxisome proliferator-activated receptor alpha mediates the adaptive response to fasting.. J Clin Invest.

[5] Guan HP, Goldstein JL, Brown MS, Liang G (2009). Accelerated fatty acid oxidation in muscle averts fasting-induced hepatic steatosis in SJL/J mice.. J Biol Chem.

[6] Sesti G, Federici M, Hribal ML, Lauro D, Sbraccia P (2001). Defects of the insulin receptor substrate (IRS) system in human metabolic disorders.. FASEB J.

[7] Previs SF, Withers DJ, Ren JM, White MF, Shulman GI (2000). Contrasting effects of IRS-1 versus IRS-2 gene disruption on carbohydrate and lipid metabolism in vivo.. J Biol Chem.

[8] Ruiz-Alcaraz AJ, Liu HK, Cuthbertson DJ, McManus EJ, Akhtar S (2005). A novel regulation of IRS1 (insulin receptor substrate-1) expression following short term insulin administration.. Biochem J.

[9] Kubota N, Kubota T, Itoh S, Kumagai H, Kozono H (2008). Dynamic functional relay between insulin receptor substrate 1 and 2 in hepatic insulin signaling during fasting and feeding.. Cell Metab.

[10] Canettieri G, Koo SH, Berdeaux R, Heredia J, Hedrick S (2005). Dual role of the coactivator TORC2 in modulating hepatic glucose output and insulin signaling.. Cell Metab.

[11] Ide T, Shimano H, Yahagi N, Matsuzaka T, Nakakuki M (2004). SREBPs suppress IRS-2-mediated insulin signalling in the liver.. Nat Cell Biol.

[12] Haeusler RA, Accili D (2008). The double life of Irs.. Cell Metab.

[13] Masternak MM, Al-Regaiey KA, Del Rosario Lim MM, Jimenez-Ortega V, Panici JA (2005). Effects of caloric restriction on insulin pathway gene expression in the skeletal muscle and liver of normal and long-lived GHR-KO mice.. Exp Gerontol.

[14] DiRusso CC, Li H, Darwis D, Watkins PA, Berger J (2005). Comparative biochemical studies of the murine fatty acid transport proteins (FATP) expressed in yeast.. J Biol Chem.

[15] Cazanave SC, Gores GJ (2010). Mechanisms and clinical implications of hepatocyte lipoapoptosis.. Clin Lipidol.

[16] Milger K, Herrmann T, Becker C, Gotthardt D, Zickwolf J (2006). Cellular uptake of fatty acids driven by the ER-localized acyl-CoA synthetase FATP4.. J Cell Sci.

[17] Watkins PA (2008). Very-long-chain acyl-CoA synthetases.. J Biol Chem.

[18] Stahl A, Evans JG, Pattel S, Hirsch D, Lodish HF (2002). Insulin causes fatty acid transport protein translocation and enhanced fatty acid uptake in adipocytes.. Dev Cell.

[19] Hirsch D, Stahl A, Lodish HF (1998). A family of fatty acid transporters conserved from mycobacterium to man.. Proc Natl Acad Sci U S A.

[20] Sandoval A, Fraisl P, Arias-Barrau E, Dirusso CC, Singer D (2008). Fatty acid transport and activation and the expression patterns of genes involved in fatty acid trafficking.. Arch Biochem Biophys.

[21] Falcon A, Doege H, Fluitt A, Tsang B, Watson N (2010). FATP2 is a hepatic fatty acid transporter and peroxisomal very long-chain acyl-CoA synthetase.. Am J Physiol Endocrinol Metab.

[22] Doege H, Baillie RA, Ortegon AM, Tsang B, Wu Q (2006). Targeted deletion of FATP5 reveals multiple functions in liver metabolism: alterations in hepatic lipid homeostasis.. Gastroenterology.

[23] Hubbard B, Doege H, Punreddy S, Wu H, Huang X (2006). Mice deleted for fatty acid transport protein 5 have defective bile acid conjugation and are protected from obesity.. Gastroenterology.

[24] Doege H, Grimm D, Falcon A, Tsang B, Storm TA (2008). Silencing of hepatic fatty acid transporter protein 5 in vivo reverses diet-induced non-alcoholic fatty liver disease and improves hyperglycemia.. J Biol Chem.

[25] Bechmann LP, Gieseler RK, Sowa JP, Kahraman A, Erhard J (2010). Apoptosis is associated with CD36/fatty acid translocase upregulation in non-alcoholic steatohepatitis.. Liver Int.

[26] Mitsuyoshi H, Yasui K, Harano Y, Endo M, Tsuji K (2009). Analysis of hepatic genes involved in the metabolism of fatty acids and iron in nonalcoholic fatty liver disease.. Hepatol Res.

[27] Auinger A, Valenti L, Pfeuffer M, Helwig U, Herrmann J (2010). A promoter polymorphism in the liver-specific fatty acid transport protein 5 is associated with features of the metabolic syndrome and steatosis.. Horm Metab Res.

[28] Kohli R, Kirby M, Xanthakos SA, Softic S, Feldstein AE (2010). High-fructose, medium chain trans fat diet induces liver fibrosis and elevates plasma coenzyme Q9 in a novel murine model of obesity and nonalcoholic steatohepatitis.. Hepatology.

[29] Metcalfe LD (1960). Gas chromatography of unesterified fatty acids using polyester columns treated with phosphoric acid.. Nature.

[30] Jandacek RJ, Heubi JE, Tso P (2004). A novel, noninvasive method for the measurement of intestinal fat absorption.. Gastroenterology.

[31] Kohli R, Pan X, Malladi P, Wainwright MS, Whitington PF (2007). Mitochondrial reactive oxygen species signal hepatocyte steatosis by regulating the phosphatidylinositol 3-kinase cell survival pathway.. J Biol Chem.

[32] Koppe SW, Sahai A, Malladi P, Whitington PF, Green RM (2004). Pentoxifylline attenuates steatohepatitis induced by the methionine choline deficient diet.. J Hepatol.

[33] Kusunoki M, Tsutsumi K, Inoue Y, Hara T, Miyata T (2004). Lipoprotein lipase activator NO-1886 improves fatty liver caused by high-fat feeding in streptozotocin-induced diabetic rats.. Metabolism.

[34] Feldstein AE, Werneburg NW, Canbay A, Guicciardi ME, Bronk SF (2004). Free fatty acids promote hepatic lipotoxicity by stimulating TNF-alpha expression via a lysosomal pathway.. Hepatology.

[35] Cherrington AD (1999). Banting Lecture 1997. Control of glucose uptake and release by the liver in vivo.. Diabetes.

[36] Schaffer JE, Lodish HF (1994). Expression cloning and characterization of a novel adipocyte long chain fatty acid transport protein.. Cell.

[37] Heinzer AK, Watkins PA, Lu JF, Kemp S, Moser AB (2003). A very long-chain acyl-CoA synthetase-deficient mouse and its relevance to X-linked adrenoleukodystrophy.. Hum Mol Genet.

[38] Hui TY, Frohnert BI, Smith AJ, Schaffer JE, Bernlohr DA (1998). Characterization of the murine fatty acid transport protein gene and its insulin response sequence.. J Biol Chem.

[39] Kerouz NJ, Horsch D, Pons S, Kahn CR (1997). Differential regulation of insulin receptor substrates-1 and -2 (IRS-1 and IRS-2) and phosphatidylinositol 3-kinase isoforms in liver and muscle of the obese diabetic (ob/ob) mouse.. J Clin Invest.

[40] Guo S, Copps KD, Dong X, Park S, Cheng Z (2009). The Irs1 branch of the insulin signaling cascade plays a dominant role in hepatic nutrient homeostasis.. Mol Cell Biol.

[41] Taniguchi CM, Ueki K, Kahn R (2005). Complementary roles of IRS-1 and IRS-2 in the hepatic regulation of metabolism.. J Clin Invest.

[42] Rojas FA, Hirata AE, Saad MJ (2001). Regulation of IRS-2 tyrosine phosphorylation in fasting and diabetes.. Mol Cell Endocrinol.

[43] Simmgen M, Knauf C, Lopez M, Choudhury AI, Charalambous M (2006). Liver-specific deletion of insulin receptor substrate 2 does not impair hepatic glucose and lipid metabolism in mice.. Diabetologia.

[44] Shimomura I, Matsuda M, Hammer RE, Bashmakov Y, Brown MS (2000). Decreased IRS-2 and increased SREBP-1c lead to mixed insulin resistance and sensitivity in livers of lipodystrophic and ob/ob mice.. Mol Cell.

[45] White MF (2003). Insulin signaling in health and disease.. Science.

[46] Gupta NA, Mells J, Dunham RM, Grakoui A, Handy J (2010). Glucagon-like peptide-1 receptor is present on human hepatocytes and has a direct role in decreasing hepatic steatosis in vitro by modulating elements of the insulin signaling pathway.. Hepatology.

[47] Luiken JJ, Koonen DP, Willems J, Zorzano A, Becker C (2002). Insulin stimulates long-chain fatty acid utilization by rat cardiac myocytes through cellular redistribution of FAT/CD36.. Diabetes.

[48] Standaert ML, Kanoh Y, Sajan MP, Bandyopadhyay G, Farese RV (2002). Cbl, IRS-1, and IRS-2 mediate effects of rosiglitazone on PI3K, PKC-lambda, and glucose transport in 3T3/L1 adipocytes.. Endocrinology.

[49] Smith U, Gogg S, Johansson A, Olausson T, Rotter V (2001). Thiazolidinediones (PPARgamma agonists) but not PPARalpha agonists increase IRS-2 gene expression in 3T3-L1 and human adipocytes.. FASEB J.

[50] Gunton JE, Delhanty PJ, Takahashi S, Baxter RC (2003). Metformin rapidly increases insulin receptor activation in human liver and signals preferentially through insulin-receptor substrate-2.. J Clin Endocrinol Metab.

